# Clinical pattern of synthetic cannabinoids users in Upper Egypt: cross-sectional study

**DOI:** 10.1186/s43045-022-00188-y

**Published:** 2022-03-30

**Authors:** Wafaa M. Abdelmoneim, Nagwa M. Ghandour, Mohamed Fawzy, Marwa Kh. Mohammed, Abdelrahman G. Ramadan, Nora Z. Abdellah

**Affiliations:** 1 Forensic Medicine and Clinical Toxicology Department, Faculty of Medicine, Assiut University, Assiut, Egypt; 2 Neurology and Psychiatry Department, Faculty of Medicine, Assiut University, Assiut, Egypt; 3 Assiut University Hospital, Assiut, Egypt

**Keywords:** Synthetic cannabinoids, Abuse, Violence, Recreational use, New psychoactive

## Abstract

**Background:**

There is an expanding use of new psychoactive substances containing synthetic cannabinoids in the last years. This study was conducted to identify the epidemiologic data of acute and chronic toxicity by synthetic cannabinoids in Upper Egypt patients.

**Results:**

All cases included in the presenting study were fifty males. Most users of synthetic cannabinoids were in the adolescence and middle age group (15–< 35) representing 68%. Curiosity was the most common motivator for using synthetic cannabinoids. Alteration of perception was reported in 68% of subjects after synthetic cannabinoids use. Additionally, dizziness, loss of consciousness, convulsion, and panic attacks were also reported. Cardiovascular adverse effects experienced by users were palpitations (76%) and chest pain (12%). Half of included subjects (50%) reported financial problems and about one-third (32%) got involved in domestic violence. Abnormal routine laboratory findings that were found in included cases were in the form of 12% anemia, 10% leukocytosis, and 6% leucopenia. Also, liver and kidney functions were elevated in 8% and 4% of the cases, respectively. While 22% and 4% of cases were positive for hepatitis C and HIV respectively.

**Conclusions:**

This study can be concluded that adolescence are the most common users of SCs; neuro-psychiatric and cardiovascular side effects were the most experienced by subjects. Violence in many forms, especially domestic violence, was associated with synthetic cannabinoids abuse.

**Trial registration:**

Registered in clinical trial under name syntheticcannabinoidsAssiut and ID NCT03866941 and URL.

## Background

There is expanding abuse of new psychoactive substances (NPS) in the last 2 decades resulting in acute toxicity, hospital admissions, and even deaths [49]. Those NPS are usually unregulated, because they are modified molecules designed to produce similar effects to those produced by illegal drugs [42].

Synthetic cannabinoids (SCs) are a class of designer drugs which mimic the psychoactive properties of Δ9-tetrahydrocannabinol (Δ9-THC), the active principle of cannabis plant (marijuana), binding the same receptors CB_1_ and CB_2_. Those SCs were produced at first as pharmaceutical agents, to take advantage of the clinical properties of marijuana and Δ9-THC [54]. Unfortunately, these compounds were abused recently to escape drug regulation laws and regular cannabinoid blood tests especially by young adults [8, 20].

Most SCs are available in the form of spice. The term “spice” refers to a group of psychoactive products, which contain a herbal mixture, with the primary active ingredients (one or more) being cannabinoid receptor agonists sprayed onto the herbal matrix [2, 48]. Although spice packages’ labels obviously state “not for human utilization,” or “ just as aromatherapy,” clinical toxicologists reported that these preparations are widely abused [39].

Some spice products have been reported to contain other compounds such as amides of fatty acids, vitamin E to mask detection of SCs, flavors, preservatives, sympathomimetic agents such as clenbuterol, benzodiazepines such as phenazepam, o-desmethyltramadol (tramadol’s active metabolite), μ-opioid receptor agonist (mitragynine), and other potent opioids (C [2, 19, 31]).

Most SCs products on the market have never been tested in vivo, even in animal models, and only a limited knowledge can be obtained from international medical databases, so their physiologic and pharmacologic effects are unpredictable [18]. Also, despite the growing concerns about the potential negative public health consequences of SC use, current surveillance of SC hazards is limited [41].

This work was conducted to

1.Evaluation of the epidemiologic data of acute and chronic toxicity by SCs in patients presenting at Assiut university hospital and Psychiatric Hospital of ministry of health using medical records and clinical examination

2.Describe the relationship between different types of violence and SCs abuse

## Methods

This is a descriptive cross-sectional study.

### Patients

All eligible cases of abusers admitted to Assiut university hospital and Psychiatric Hospital of ministry of health in the period from October 2019 to January 2021 were evaluated for inclusion and exclusion criteria.

#### Inclusion criteria

- Fifty cases with the primary diagnosis of acute or chronic SCs toxicity.
- Subjects willing and able to comply with the study procedures and provide written informed consent to participate in the study.

#### Exclusion criteria

- Patients with a history of any chronic disease (renal, cardiac, hepatic, and autoimmune).
- Refusal to participate in the study.

A fifty male case were evaluated by detailed history and through fulfilling a questionnaire of substance misuse (modified form) [23] (Appendix 1) which include:

1. Epidemiological data (age, sex, residence, occupation, route of abuse, cause of abuse, route of administration and smoking)
2. Symptoms and signs (seizures, psychosis, hallucinations).
3. Injuries and their relation to violence (type of wound, type of instrument, number of injuries, external or internal injuries and either assailant or victim).

#### Method

Investigations done for included cases:

- Liver functions (AST and ALT)
- Kidney function (urea and creatinine)
- CBC, glucose level
- Urine drug screen

### Statistical analysis

Data entry and analysis carried out using SPSS version 19. Descriptive statistics were done in the form of frequencies, mean and standard deviation then analytic statistics were done as chi square, independent sample *t* test, one-way ANOVA, and correlations tests. *P* values were considered significant when equal to or less than 0.05.

### Research ethics

- Reviewing the proposal was carried out before starting data collection via the Ethical Review Committee of Faculty of Medicine- Assiut university.
- Privacy and confidentiality of all the data were assured.
- Written consent was obtained from subjects or their caregiver (less than 18 years old) who agreed to participate in the study. Providing adequate information about participation to enable subjects to understand the consequences of participating and to reach a totally informed, considered, and willfully given decision about whether to participate, without any stress or coercion. The consent form in Arabic and English in Appendix 2.

## Results

The total number of synthetic cannabinoid users who were included in the study was fifty male cases in the period from October 2019 to January 2021 (cases admitted to Neuro-Psychiatric Assiut University hospital and Psychiatric hospital of the ministry of health in Assiut). This number of cases came in line with the decline of legally reported cases when comparing 2018 with 2019 according to the Narcotics control Bureau in Assiut Governorate. In 2018, four cases of drug trafficking were reported in the Drug Enforcement Administration (DEA) of Upper Egypt and a single case of drug trafficking reported in narcotics bureau of the security directorate in addition to 5 cases of trafficking and abuse reported in police stations. In contrast, no cases were reported in DEA, narcotics bureau or in police stations in 2019. Additionally, there was a dropout in cases in the period from 15th March to 1st September 2020 due to COVID-19 pandemic.

### Epidemiological characteristics

Table 1 showed age distribution among the studied abusers of synthetic cannabinoids. The age ranged from 15 to 55 years. The mean age was 29.48 ± 7.89 years old. Most of the abusers were in adolescence and middle age group (15–< 35) representing 68% of the studied cases, followed by the age group (35–< 45) representing 28% of the studied cases. The least percentage was in the age group [45–55] representing only 4% of the studied cases. As regards residence distribution, 56% of the studied cases came from rural areas and 44% of them were from urban areas. Regarding occupation, 40% of abusers were merchants, while free workers represented 34% of the studied cases, followed by drivers 20% of the studied cases, and then farmers 6% of the studied cases. Nearly more than half of abusers (56%) had secondary technical education. High education (institutional), secondary-general, preparatory, primary schools, and illiteracy represented 2%, 4%, 22%, 10%, and 6% respectively. Single individuals represented 54% of abusers, while married were 40% of the studied cases, followed by divorced 6% of the studied cases. It was found that 100% of the studied subjects are smokers with the mean pack per year smoking index 27.5 ± 17.7 and the mean age of starting smoking 13.8 ± 2.9 years old.

**Table 1:** Epidemiological characteristics of the 50 studied abusers of synthetic cannabinoids

	Number ***N*** = 50	Percentage (%)
**Age groups**		
15–< 25	17	34%
25–< 35	17	34%
35–< 45	14	28%
45–55	2	4%
**Mean age of synthetic cannabinoids substance abusers in years ± SD =** 29.48 ± 7.89		
**Residence**		
Rural	28	56%
Urban	22	44%
**Occupation**		
Merchant	20	40%
Worker	17	34%
Driver	10	20%
Farmer	3	6%
**Educational level**		
Institutional	1	2%
Secondary- General	2	4%
Secondary- Technical	28	56%
Preparatory	11	22%
Primary	5	10%
Illiterate	3	6%
**Marital status**		
Single	27	54%
Married	20	40%
Divorced	3	6%
**Smoking**		
Non smoker	0	0%
Less than one pack/day	6	12%
Pack and half/day	18	36%
More than pack and half/day	26	52%

Mean pack per year smoking index of studied abusers ± SD = 27.5 ± 17.7

Mean age of starting smoking in years ± SD = 13.8 ± 2.9

### Abuse data

Table 2 demonstrated that 60% of the studied subjects were strox users, 34% of them were strox and voodoo users, and only 6% of them were voodoo users. Regarding the place of administering the substance, nearly two thirds (64%) of subjects reported street as place for substance abuse. Both home and street were reported by 18% of abusers followed by home only 10% of the abusers and work 8% of the abusers. In view of the route of administration, all the studied subjects administered abused material via smoking cigarettes. Also, regarding the age of starting substance abuse and that of starting strox abuse, their mean values were 17.5 ± 4.5 and 28 ± 8 years old, respectively. Multiple time users represented 64% of subjects while 36% of cases reported single time administration. Experiencing relapse in a trial to abstain was found in 10% of multiple time abusers and half of them reported the need to increase the dose to get the initial effects. Regarding motivations for using strox, curiosity was the most reported cause 34% followed by friends’ suggestion 32%. Availability of strox, sadness, money shortage, need to increase power to work, and non-availability of his usual substance represented 16%, 12%, 2%, and 2% respectively.

**Table 2:** Abuse data

	Number ***N*** = 50	Percentage (%)
**Name of synthetic cannabinoids abused substance**		
Strox	30	60%
Strox and voodoo	17	34%
Voodoo	3	6%
**Place of administration**		
Street	32	64%
Home and street	9	18%
Home	5	10%
Work	4	8%
**Number of uses**		
Multiple	32	64%
Single	18	36%
**Occurrence of relapses in case of multiple users (*N*** **= 32)**		
No	29	90%
Yes	3	10%
**The need to increase the dose to get the same effect in case of multiple users (*N*** **= 32)**		
Yes	16	50%
No	16	50%
**Route of taking substance**		
Inhalational (smoking as cigarettes)	50	100%
**Cause of abuse**		
Curiosity	17	34%
Friends	16	32%
Availability of synthetic cannabinoid	8	16%
Sadness	6	12%
Money shortage	1	2%
Increase power to work	1	2%
Non availability of his usual substance	1	2%

Mean age of starting substance abuse in years ± SD = 17.5 ± 4.5

Mean age of starting synthetic cannabinoid abuse in years ± SD = 28 ± 8

### Past medical history

Table 3 illustrated the past medical history of the included 50 abusers. Regarding previous admission to psychiatric hospitals, 28% of the patients were admitted as inpatient cases and only 4% were admitted in ICU because of drugs overdose, whereas 68% of the patients were not admitted at all. Additionally, 4% of the subjects exhibited a psychiatric illness history of depression whereas 96% of them did not experience any psychiatric illness. Moreover, family history of substance abuse was positive in 38% of the cases and was negative in the remaining 62%.

**Table 3:** Past medical history

	Number ***N*** = 50	Percentage (%)
**Previous admission to psychiatric hospitals**		
No	34	68%
Yes, as inpatient	14	28%
Yes, as ICU	2	4%
**Psychiatric history**		
No	48	96%
Yes	2	4%
**Family history of abuse substances**		
No	31	62%
Yes	19	38%

### Symptoms experienced by the included cases following SCs use

Table 4 exhibited that the mostly reported neuro-psychiatric symptom was dizziness or drowsiness since they were experienced by 68% of the included cases, followed by 62% reporting hallucinations (either visual and auditory or auditory only). While 22% of the included patients experienced headache, 8% experienced seizures and only 4% experienced agitation and irritability. Panic attacks and fear of death was reported in 26% of the included cases and psychosis was found only in one case. Cardiovascular symptoms were also of important concern where palpitations were described by 76% of the studied cases and chest pain in 12% of them. Moreover, gastrointestinal symptoms in the form of nausea and vomiting were also manifested in almost quarter (24%) of them. Finally, other symptoms in the form of blurring of vision and red eye were described by 88% of the studied cases and although 20% of included users suffered from withdrawal manifestations, 4% of the included cases did not experience any effects at all.

**Table 4:** Symptoms of toxicity or withdrawal experienced after SCs use

	Number ***N*** = 50	Percentage (%)
**Neuro-psychiatric effects**		
**Dizziness/Drowsiness**	34	68%
**Headache**	11	22%
**Seizures**	4	8%
**Irritability/agitation**	2	4%
**Auditory and visual hallucination**	29	58%
**Auditory only hallucination**	2	4%
**Panic attack (fear of death)**	13	26%
**Psychosis**	1	2%
**Cardiovascular effects**		
**Palpitation**	38	76%
**Chest Pain**	6	12%
**Gastrointestinal effect**		
**Nausea and vomiting**	12	24%
**Others**		
**Blurred vision and red eye**	44	88%
**Withdrawal manifestation**	10	20%
**No symptoms were experienced**	2	4%

### First dose and effect duration

Table 5 showed that the mean number of breaths received as a first dose by the included abusers was 2.46 ± 0.86 breaths and the effect duration in first exposure was 32.9 ± 23.8 min. Moreover, there is a highly statistically significant positive correlation between number of breaths received and the effect duration (*P* value of less than 0.001). Also, regarding the maximum number of cigarettes received per day on the long run was 4 ± 2.7.

**Table 5:** First dose and effect duration

	Mean ± SD	***P*** value
**First dose received (Subjective number of breaths)**	2.46 ± 0.86 breaths	0.001* >
**Duration of effect in first exposure**	32.9 ± 23.8 min	
**Maximum dose**	4 ± 2.7 cigarettes/day	
**Range of first dose duration**: 7–90 min		

*P* value is significant at ≤ 0.05

### History of injuries, violence, and problems associated with the drug abuse

Table 6 demonstrated that half of them included subjects (50%) reported financial problems and about one third (32%) got involved in domestic violence. Additionally, psychiatric problems were reported in 8% of the cases and legal problems in 6% of the cases, whereas only 4% of the cases reported health problems. Furthermore, 14 cases out of the 50 cases were involved in injurious events, half of them were victims, and the other half were assailants under the SCs influence. On exploring the injury types as victim, they came out to be mostly cut wounds (suicidal attempts associated with impulsivity and the urge feeling that he needs to harm himself and feeling of relief after doing that) representing 86% of the total injuries and only 14% in the form of fracture and was caused by others. As regards injuries as an assailant, also cut wounds were the most common being 44% of total injuries. But lacerated wounds, firearm, stab wounds, and bruises were all inflicted in an equal percent of cases representing 14% for each. There was also a legal involvement in 15 cases out of 50. The breakdown of those 15 cases came as follows: 20% of them were involved in drug dealing and became suppliers themselves, 27% of them were legally considered as assailants, 40% of them were accused of abusing, and 13% of them involved legally in both; assault and drug dealing.

**Table 6:** Injuries, violence, and problems associated with the drug abusing

	**Total number**		**Percentage (%)**	
**Subjective problems associated with the substance abuse (*N*** **= 50)**				
**Financial**	**25**		**50%**	
**Domestic violence**	**16**		**32%**	
**Psychiatric**	**4**		**8%**	
**Lawful**	**3**		**6%**	
**Health**	**2**		**4%**	
**Injuries reported by the users (N=14)**				
	***AS a victim*** **(*N*** **=** ***7*)**		***As an assailant*** **(*N*** **=** ***7*)**	
	***N***	**%**	***N***	**%**
**Cut wounds**	**6**	**86%**	**3**	**44%**
**Lacerated**	**-**	**-**	**1**	**14%**
**Firearm**	**-**	**-**	**1**	**14%**
**Stab**	**-**	**-**	**1**	**14%**
**Bruises**	**-**	**-**	**1**	**14%**
**Fractures**	**1**	**14%**	**-**	**-**
**Legal causes for which abusers arrested (*N*** **= 15)**				
**Abusing**	**6**		**40%**	
**Assault**	**4**		**27%**	
**Supplier**	**3**		**20%**	
**Supplier and assault**	**2**		**13%**	

### Urine drug screen

Table 7 illustrated the results of urine drug screening in which opioids were the more drug to be detected (30% of studied cases) followed by both cannabinoids and opiates in 18% of the cases. Furthermore, cannabinoids only were detected in 10% of the subjects and tramadol alone was detected in 12% of the subjects, yet both tramadol and opiates were detected in 4%. Another 4% of subjects were positive for mixture of drugs (cannabinoids in addition to tramadol and opiates). Lastly, 10% of the included cases were negative for any dugs and the test was not conducted in 12% of cases due to their refusal.

**Table 7:** Results of urine drug screen

	Number ***N*** = 50	Percentage (%)
**Positive samples**	**39**	**78%**
**For opiates**	**15**	**30%**
**For cannabinoids and opiates**	**9**	**18%**
**For Tramadol**	**6**	**12%**
**For cannabinoids**	**5**	**10%**
**For cannabinoids, tramadol, and opiates**	**2**	**4%**
**For tramadol and opiates**	**2**	**4%**
**Negative samples**	**5**	**10%**
**Not done (refusing)**	**6**	**12%**

### Laboratory investigations

Table 8 showed the mean values of routine investigations conducted at the time of presentation and the presented abnormality. Complete blood count (CBC) showed that the mean hemoglobin concentration in all included cases was 14.2 ± 1.8 g/dl. Furthermore, on comparing individual results with the standard reference range of hemoglobin, it came out that 6 patients were considered to have anemia representing 12% of the included subjects. Also, the mean count for white blood cells was 7.3 ± 2.4 × 10^3^ per microliter and individual results included 5 cases (10%) with leucocytosis in addition to 3 cases (6%) with leucopenia. Additionally, mean value for platelet count was 288 ± 64 × 10^3^ per microliter and none of the included cases had neither thrombocytosis nor thrombocytopenia. Moreover, liver enzymes were abnormally elevated in 4 cases representing 8% and the mean value for ALT was 29.8 ± 26.5 and for AST was 33.1 ± 32.2 IU/L. Renal functions were estimated through measuring urea and creatinine levels and their mean concentrations were 14.5 ± 6.4 and 73.6 ± 27.4 mg/dl respectively, which were abnormal in only 2 cases representing 4%. Blood glucose levels were also measured and exhibited no abnormalities with mean concentration of 5.1 ± 0.8 mmol/l. Furthermore, on exploring the serology result, 22% had hepatitis C virus and 4% had HIV.

**Table 8:** Results of laboratory investigations of the abusers

	**Mean ± SD**	
**Complete Blood Count (CBC)**		
**Hemoglobin (g/dl)**	14.2 ± 1.8	
**White blood cells (/μL)**	7.3 ± 2.4 × 10^3^	
**Platelets (/μl)**	288 ± 64 × 10^3^	
**Liver function**		
**ALT (IU/L)** Normal range (7–56 IU/L)	29.8 ± 26.5	
**AST (IU/L)** Normal range (5–40 IU/L)	33.1 ± 32.2	
**Renal function**		
**Urea (mg/dl)** Normal range (5–20 mg/dl)	14.5 ± 6.4	
**Creatinine (μmol/L)** Normal range (60 to 110 μmol/L)	73.6 ± 27.4	
**Glucose**		
**Random Blood glucose (mmol/l)** Normal range (3.9–5.6 mmol/l)	5.1 ± 0.8	
Abnormality	Number of cases	Percentage
**Cases with anemia**	6	12%
**Cases with leucocytosis**	5	10%
**Cases with leukopenia**	3	6%
**Cases with elevated liver function**	4	8%
**Cases with elevated Kidney function**	2	4%
**Serology**		
**Positive Hepatitis C**	11	22%
**Positive HIV**	2	4%

## Discussion

SCs abuse has gained great concern due to its increasing popularity and lack of awareness of its hazards as it is regarded by abusers as a natural plant owing to its appearance. Furthermore, insufficient information is known about the long-term consequences of chronic SCs misuse [9].

The present descriptive study was conducted from October 2019 to January 2021, and it aimed at evaluation of the epidemiologic data of acute and chronic toxicity by SCs in patients presenting at Assiut university hospital and Psychiatric Hospital of ministry of health using medical records and clinical examination to assess the personal experiences and how the participants interpret their experience with SCs use. Also, to describe relationship between different types of violence and SCs abuse.

Regarding the epidemiological data, the current study showed that most users of synthetic cannabinoids were in the adolescence and middle age group (15–< 35) representing 68% of the studied cases, and this in accordance with surveys of substance use in USA where it has found that SCs use is more concentrated in adults aged (19–28 years) [27] and as well with the finding of Loeffler et al. [29] who reported that abuse of SCs tended to peak in the adolescence (late teens and early twenties). All cases included in the presenting study were males and this comes in agreement with the results of [5, 53]. This can be explained here by fear from addiction stigma especially with females in our society. Most of the abusers in this study had 12 or less years of education and this is the same found in the study of Cohen et al. [13] where SC users had fewer years of study than others users.

While Castellanos and Thornton [11] and Stogner [47] found no associations between SCs use and age, marital status, athlete status, and employment.

All the included subjects in the current study were smokers. Cohen et al. [13] explained that early onset of smoking cigarettes is usually associated with high incidence of substance abuse. Also, Palamar and Acosta [37] and Clayton et al. [12] reported significant association between smoking regularly and using SCs.

Among the studied SCs users, it was found that multiple time users represented 63% of them. Multiple users used SCs as a replacement or substitute for another substance because of its unavailability or its price. This comes with agreement with Baggio et al. [4] in the survey of Swedish military recruits which revealed that SCs use tends to be unmaintained. Frequency of use appears to be restricted to a few numbers of times. Rather than being used on a long-term basis. In the existing study, participants reported that the cause of discontinuation is that their regular substance produces more powerful and longer effect, others reported the undesired effect (fear of death and panic attack). The fact that mentioned by those users is that their own experience of natural cannabis seems to be preferred to SC in terms of both more positive effects and less negative effects, which may interpret why SC is only tried a few times then discontinued.

According to the route of administration of SCs, all cases in the current study were rolling a drug into a joint using unknown dose for smoking. This was agreed with the studies done in the USA, the UK, Australia, and Canada [3, 32]. SCs are consumed typically as cannabis products as both are not ruined by the heat generated within the cigarette. As regards the place of administering the substance, nearly two-thirds (64% of the studied group) take it in the street followed by (18%) of them taking it at home and in street, and 10%, 8% of the cases at home only and at work, respectively. However, in the study conducted by Maxwell [32], it was demonstrated that the exposure sites were in the patient’s home or another residence (unknown location and in a public area).

In the current study regarding the cause of abuse, curiosity was the most motivation for users to try SCs followed by friends. The same was found by Goode and Ben-Yehuda [21] and Loeffler et al. [29] who reported that curiosity was responsible for the spread of most other illicit drugs like marijuana in the 1930s, to lysergic acid diethylamide and glue-sniffing in the 1960s, phencyclidine in the 1970s, cocaine in the 1980s, and, most recently, methamphetamine, ecstasy, and other street drugs. Additionally, non-availability of the usual abuse substance and cost in addition to friends were reported as other motives for the use of SCs in the studies of Werse et al. [57], Barratt et al. [5], and Wagner et al. [55].

In the presenting study, concerning the age of starting addiction, the mean age of starting substance abuse was 17.5 ± 4.5 years (adolescence) and this was associated with starting to use SCs at 28 ± 8 years of age. Clayton et al. [12] suggested that early onset of addiction is associated with multiple drugs abusing and reported that early marijuana might be a risk factor for subsequent SCs use. Marijuana is usually described as being the gateway for other drug abuse [45, 51].

Half of multiple users of SCs in the current study experienced the need to increase the initial dose to get the same primary effect (tolerance). This was in agreement with many studies of Vandrey et al. [53], Panlilio et al. [38], and van Amsterdam et al. [50] reported the rapid development of tolerance with using SCs. In the existing study, regarding relapse following trials to discontinue using SCs, this was observed in only 10% of multiple users with mild withdrawal symptoms which was resolved by using cannabis or more addictive substances like opioids or heroin. Most commonly described withdrawal manifestations were headache, fatigue, insomnia, anxiety, loss of appetite, and aggressiveness. Withdrawal symptoms after cessation of SCs use were also described by [9, 46]. The presence of withdrawal syndrome associated with SCs may lead users to seek more addictive substances as what was found in the current study.

Considering past medical history, it was important to exclude the presence of a previous psychiatric history of the cases to detect the psychiatric effects of SCs. In the present study, psychiatric illness history was exhibited in 4% of the subjects, whereas 96% did not experience any psychiatric illness. However, admission of the cases to psychiatric hospitals was to manage addiction and not to treat psychiatric disorders.

In the context of family history of addiction, it was positive in 28% of the cases mostly for cannabis and was negative in the remaining 62%. Family history became an important factor to evaluate in substance misuse as it was found that it influences the outcome of the cases as patients with positive family history are associated with more incidence of comorbid psychiatric disorders, co-morbid substance dependencies, severe antisocial behavior, and greater medical problems than those with no family history [14].

Concerning the symptoms experienced in the included cases following SCs abuse, most SCs are extraordinarily potent and effective, as a result of the full agonism of the cannabinoid receptors (CB_1_ and CB_2_) [56]. However, SCs may bind with non-cannabinoid receptor directly, such as the vanilloid type1 receptor (TRPV1) [17], or by forming heterodimers between CB_1_ receptors and D_2_ dopamine, μ-opioid, or orexin-1 receptors [26, 34]. The clinical effects of SCs might be quite unpredictable, even among subjects who have smoked the same batch of SCs [28]. The neuropsychiatric manifestations may be explained mostly as being a result of an imbalance of a number of neurotransmitter pathways and receptors. Being a highly lipophilic compound and crossing the blood-brain-barrier easily [16].

Results obtained in this study revealed that the neuropsychiatric effects experienced after SCs smoking, alteration in perception was reported by 66% of abusers. This included dissociation from reality, auditory, visual hallucinations, hyperactive thoughts, and irritability. This agreed with Bonaccorso et al. [6],who stated the psychoactive effects produced by high doses of SCs. In the study done by Castellanos et al. [10], it showed that hallucinations are five times more common to develop after consuming SCs as compared to cannabis. Additionally, dizziness or drowsiness were among the most reported CNS symptoms among users (68%). Passing out and losing consciousness were also observed. Others suffered convulsion and fell on the floor prior to blackouts (8%). Users stated having panic attacks related to the use of SCs then eventually passed out (26%). This comes in agreement with the finding of Schifano et al. [43] who reported panic attacks; thought disorganization, and agitated/excited delirium. Headache as well was described by some users after being waken up the next day of using the SCs (22%). Acute severe psychosis with delusion of grandiosity, which developed after a multiple use of SCs (third dose) utilization was found in one patient, lasted for several days, and needed hospital admission.

The major cardiovascular adverse effects experienced by abusers were palpitations (76%) and chest pain (12%). Gastrointestinal adverse effects experienced by abusers included general gastrointestinal irritations, with nausea, and vomiting (24%). This is different from the supposed action of cannabinoids as anti-emetic drugs. Opposite to the presenting finding, the most reported side effect was cardiovascular in the form of tachycardia and this is also observed in the study of Maxwell [32]. Controversies to these results Abass et al. [1] showed that the most reported effect with the use of voodoo (other commonly used synthetic cannabinoid) was the neuropsychiatric in the form of hallucination and gastrointestinal in the form of nausea and vomiting.

Withdrawal symptoms experienced by users in the presenting results after discontinuing SCs included anxiety, myalgia, insomnia, nausea, diarrhea, and irritability (20%). This was agreed with Macfarlane and Christie [30] who described these withdrawal symptoms and assisted the need for detoxification of the SCs users with similar symptoms as present in the current study with the addition of tachycardia and tremors.

The different symptoms observed after synthetic cannabinoids consumption are related to the distribution of CB_1_ receptors in the central and peripheral neurological system. Thus, CB_1_ receptors are abundant in areas associated with affective regulation (e.g., amygdala, ventral tegmental area, nucleus accumbens), and cognitive and memory functions (e.g., hippocampus, neocortex). CB_1_ receptors are also found in the brain stem, and their activation may be the cause of synthetic cannabis’ cardiovascular, respiratory, and emetic effects [24, 52].

As regarding first dose and effect duration, the participant cases reported duration of effect range from 7 to 90 min in their first use and this was subjective estimation, as well this was positively correlated to the dose received by them quantified by number of breaths or cigarettes (they could not assess the grams used). The reported range of effect in the study of Assi et al. [3] after smoking SCs was from 2 to 270 min. They also reported their need to increase the dose to obtain the same effect with the repeated use with the maximum of 4 ± 2.7 cigarettes/day although the first dose mean was only 2.46 ± 0.86 breaths with difficulty to even finish one cigarette described by all included users.

In the context of injuries and violence, fourteen cases out of the 50 cases were involved in injurious events, half of them were victims, and the other half were assailants under the SCs influence. On exploring the injury types, they were mostly cut wounds representing 65% of the total injuries. Also, (32% of studied cases) got involved in domestic violence. There was also a legal involvement in 15 cases out of 50 as follows: 40% were accused of abusing, 20% of them were involved in drug dealing and became suppliers themselves, 27% were legally considered as assailants, and 13% were involved legally in both; assault and drug dealing. The higher prevalence of psychotic symptoms, such as agitation and aggression among SC users, is an important factor. Moreover, hallucinations that were described by many SCs users also added to the violence acts observed in these cases. So, synthetic cannabinoids are thought to be linked to concerns such as aggression, self-harm, suicide, and mental illness ([40]). The marked risk of psychopathological problems and accompanying violence occurrence that is usually associated with drug abuse is generally attributed to the imbalance of some neurotransmitter pathways and receptors [44].

This coincides with the study of Clayton et al. [12], who showed that sexual violence, harming someone with a weapon, physical fighting, and carrying a weapon were all more common among SCs users. Also, Ralphs et al. [40] correlated the sharp increase in serious violence, self-harm, and suicide in England and Wiles’ prisons to the increased consumption of SCs.

SCs users included in the present study tended mostly to be poly-drug users. This is in agreement with Stogner [47] results who found that mostly all synthetic cannabinoid users have as well used tobacco, alcohol, and cannabis. Also, they used a broad variety of substances including opioids, heroin, tramadol, and benzodiazepines. Assi et al. [3] mentioned combinations to SCs ranged from two to five substances to enhance the psychedelic effects, extend the duration of effects, or resist the impulse to re-dose. Users of SCs, compared with subjects had never used SCs, had a significantly higher prevalence of using heroin, prescription opioids/sedatives, amphetamines or ecstasy, natural cannabis, hallucinogens, inhalants, and tobacco [7].

Users of SCs typically have a history of cannabis use in the study of Hu et al. [25] or actually most studies do find high rates of overlap between the two substances [5, 12, 59]. That is confirming the possibility that marijuana usage may have been a risk factor for later synthetic cannabinoid use within the study population. Indeed, researchers usually have described marijuana usage as a risk factor for later use of other illicit drugs [33, 45]. Thus, preventing marijuana usage, especially among young teenagers, may have an impact on the reduction of synthetic cannabinoids usage. The exact doses and effects of such combinations makes interpretation of the pharmacological and toxicological effects of these substances very difficult. Additionally, the incidence of seeking emergency treatment after SCs use increased in cases who took other substances with SCs, especially alcohol [58].

Laboratory routine investigations done at time of presentation revealed that abnormal routine laboratory findings were found in included cases in the form of 12% of studied cases anemia, 10% of studied cases leucocytosis, and 6% of studied cases leucopenia. Anemia is a frequent nutritional problem seen in substance use as they are not usually concerned about their nutrition and diet. Guzel et al. [22] illustrated the effect of SCs on iron metabolism resulting in subclinical anemia in SCs users. While leucopenia may be explained by immune system activation through the stimulation of CB2 receptors which are known to be activated more by SCs than cannabis. However, it is worth noting that there are many forms of synthetic cannabinoids and each may have different effects on CB receptors that may potentially interfere with hematological results [35].

Also, liver and kidney functions were elevated in 8% and 4% of the cases, respectively. Elevated liver enzymes may correspond with the presence of 22% of cases with positive serology for hepatitis C or being affected by SCs. Finally, 4% of participants were positive for HIV. Müller et al. [36] demonstrated elevation of hepatic and renal function in association with SCs use. However, the study of Abass et al. [1] found that there was not statistically significant difference in liver and kidney function tests between the SCs users. Positive serology is more associated with the injection of substances, so the incidence concluded in the presenting study related with the co-use of other injecting substances. This coincides with Dagli [15]’s screening of hepatitis and HIV viruses among users in an alcohol and drug addiction treatment center, as they noticed that infected patients were significantly higher in the persons who used opiates than other substance included SCs. He also reported zero cases of infection with the patient used SCs as the first drug.

## Conclusions

There is rapid development and wide range of new psychoactive compounds including synthetic cannabinoids. The illegally available products have different unknown ingredients. Thus, leading to unpredictability of experienced symptoms and clinical presentation. Clinicians should suspect synthetic cannabinoid usage in patients who have had a change in mental status without an apparent cause. Moreover, the abuse of these synthetic cannabinoids was associated with multiple neuro-psychiatric and physical disorders that are hazardous to the abusers and their surroundings. Its use could also contribute to violence and abnormal behavior in relation to hallucination with its catastrophic social consequences. Moreover, the incidence of these disorders was more than that of natural cannabis.

### Limitations of the study

- As there was no way to authenticate the subjective experience of users, the current research administers information on SCs as described from people who experienced them. Lived experiences, on the other hand, are critical in the creation of clinical guidance.
- The total number of cases was low, and cases were multiple drug users.
- The effects of SCs will be related to their constituent components, which vary between different SCs.
- The difficulty in estimating the exact dose received by participants.

## Data Availability

The datasets used and/or analyzed during the current study are available from the corresponding author on reasonable request.

## Abbreviations

NPS: New psychoactive substance
SCs: Synthetic cannabinoids
Δ9-THC: Δ9-tetrahydrocannabinol
CB_1_: Cannabinoid 1 receptor
CB_2_: Cannabinoid 2 receptor
DEA: Drug Enforcement Administration
CBC: Complete blood count
